# Structure-based screening and molecular dynamics of phytophytochemicals against *pseudomonas aeruginosa* quorum sensing systems

**DOI:** 10.1016/j.jgeb.2025.100603

**Published:** 2025-10-17

**Authors:** Sinethemba H. Yakobi, Uchechukwu U. Nwodo

**Affiliations:** Patho-Biocatalysis Group (PBG), Department of Biochemistry and Microbiology, University of Fort Hare, Private Bag X1314, Alice 5700, South Africa

**Keywords:** Quorum Sensing Inhibition, *Pseudomonas aeruginosa*, Phytochemicals, Baicalin, Cinnamaldehyde, Berberine

## Abstract

*Pseudomonas aeruginosa* employs quorum sensing (QS) to regulate virulence and antibiotic resistance, making QS inhibition a promising anti-infective strategy. Here, we computationally evaluated three phytochemicals—baicalin, berberine, and cinnamaldehyde—as QS inhibitors targeting LasR, RhlR, and PqsR regulators. Molecular docking revealed berberine as the most potent PqsR binder (GScore: −6.801 kcal/mol), competitively displacing the native ligand HHQ, while baicalin showed broad-spectrum inhibition of PqsR/RhlR. Cinnamaldehyde exhibited moderate LasR antagonism. Molecular dynamics (100 ns) confirmed complex stability (RMSD < 2.5 Å) and identified key interactions: berberine formed a salt bridge with PqsR Asp264, while baicalin induced allosteric helix destabilization. Pharmacokinetic profiling showed that berberine is rapidly cleared (134.7 mL/min/kg) and poses a risk of drug–drug interactions due to CYP3A4 and CYP2D6 inhibition. This makes formulation strategies or analogue design more suitable than relying on metabolic inhibition. In contrast, baicalin has very poor absorption (bioavailability score: 0.11), indicating that nanoformulation is required to improve its uptake. Cinnamaldehyde demonstrated favourable drug-likeness but required structural optimization to mitigate aldehyde reactivity. This study provides *in-silico* mechanistic support for phytochemical-mediated QS inhibition in *P. aeruginosa*, with berberine emerging as a lead candidate for further development. Our integrative approach map water displacement hotspots in PqsR (GIST) and detect a baicalin-linked distal helix perturbation (DSSP) consistent with allostery, and bridges computational prediction and therapeutic design, offering new strategies to combat antimicrobial resistance through virulence attenuation.

## 1 Introduction

*Pseudomonas aeruginosa* (*P. aeruginosa*) opportunistic pathogen, notorious for its multidrug resistance and recalcitrant biofilm formation in chronic infections.^1^, ^2^ Central to its pathogenicity is a hierarchical quorum sensing (QS) cascade comprising three transcriptional regulators—LasR, RhlR, and PqsR—that collectively orchestrate the temporal expression of virulence factors.^3^ LasR initiates this cascade by activating genes for elastase and the transcription of *rhlR*.^4^ RhlR, in turn, governs pyocyanin biosynthesis and biofilm maturation,^5^ while PqsR mediates alkyl-quinolone signalling pivotal for iron chelation and oxidative stress resilience, essential for long-term persistence.^1^ Although synthetic QS inhibitors such as furanone C-30 have demonstrated efficacy, their clinical translation remains constrained by resistance emergence and off-target toxicity.^6^, ^7^ In contrast, phytochemicals represent a structurally diverse and evolutionarily honed chemical space with lower resistance potential.^8^, ^9^ Phytochemicals like those from *Carica papaya* have demonstrated broad anti-infective and cytotoxic properties,^10^ reinforcing the potential of plant-derived compounds as alternatives to conventional antibiotics.^11^ However, most prior studies have relied on static docking approaches and have not resolved the dynamic, water-mediated, or allosteric interactions critical for understanding true binding efficacy.^12^ This study aims to address these gaps through an integrative *in silico* approach combining molecular docking, explicit solvent molecular dynamics (100 ns), and pharmacokinetic profiling. We benchmark the QS modulatory potential of three structurally distinct phytochemicals—Baicalin (flavonoid), Berberine (alkaloid), and Cinnamaldehyde (phenylpropanoid)—against canonical QS receptors LasR, RhlR, and PqsR. Selection was guided by their reported anti-virulence activity identified, Baicalin disrupts biofilm formation (IC_50_ 20 μM),^13^ Berberine inhibits PqsR-mediated signalling,^14^ and Cinnamaldehyde reduces pyocyanin production.^15^ Their use at physiologically relevant concentrations (10 μM) reflects clinically achievable tissue levels.^8^, ^16^, ^17^ To evaluate the binding affinities of the target phytochemicals, we established quantitative benchmarks using structurally characterized QS inhibitors with experimentally validated efficacy. For PqsR inhibition, we referenced M64 (a synthetic antagonist that disrupts DNA binding and reduces virulence gene expression), which consistently demonstrates docking scores of −6.5 kcal/mol in AutoDock studies.^18^ The LasR inhibitor furanone C-30 known to suppress elastase production and biofilm formation − served as our LasR benchmark, with typical docking energies of −7.8 kcal/mol.^19^ For RhlR, was employed homology models docked with *meta*-bromo-thiolactone (mBTL), a synthetic analogue showing consistent binding near −7.0 kcal/mol.^20^ Furthermore, the CYP3A4/2D6 inhibition profile of Berberine, though a potential liability, may be mitigated by P-glycoprotein-mediated tissue targeting. The allosteric modulation of PqsR by Berberine suggests a promising strategy for combinatorial anti-QS therapy.^21^ Studies showed that RMSD comparison with crystallographic poses (RMSD < 2 Å) ensured protocol validation,^22^, ^23^ and to assess specificity, phytochemicals would be screened against human HSP90 and *P. aeruginosa* efflux proteins, retaining only those with > 5 kcal/mol selectivity for QS targets.^3^, ^24^ Altogether, this work offers mechanistic insight into phytochemical-QS receptor interactions, expanding the rational design space for anti-virulence therapeutics targeting *P. aeruginosa*.

## 2 Methods

### 2.1 Phytochemical selection

Three phytochemicals—Baicalin (CID: 64982), Berberine (CID: 2353), and Cinnamaldehyde (CID: 637511)—were selected via a multi-tiered screening strategy combining database mining, bioactivity validation, and drug-likeness assessment. Selection criteria included reported quorum sensing (QS) inhibitory effects in Gram-negative pathogens, scaffold diversity (flavonoid, alkaloid, aldehyde), and adherence to drug-likeness thresholds (molecular weight < 500 Da; LogP < 5). Phytochemicals were prioritized based on experimental evidence shown by.^3^, ^14^, ^15^ Molecules flagged with PAINS alerts or synthetic analogues with ambiguous bioactivity profiles were excluded.

### 2.2 ADME prediction

SwissADME and pkCSM were used for early-phase pharmacokinetic screening. Baicalin exhibited two Lipinski violations (MW = 446.36; HBD = 6), while Berberine triggered a Brenk alert due to its quaternary nitrogen. Cinnamaldehyde presented no violations across filters. Notably, Berberine was predicted to be a P-glycoprotein (P-gp) substrate and a dual inhibitor of CYP3A4 and CYP2D6, whereas Baicalin and Cinnamaldehyde showed no major cytochrome inhibition. Bioavailability scores were 0.55 for Berberine and Cinnamaldehyde, and 0.11 for Baicalin. pkCSM-predicted parameters further supported in vivo relevance, with all phytochemicals exhibiting volume of distribution (V_D_) values > 0.6 L/kg, suggesting favourable tissue penetration.^23^

#### 2.2.1 Protein structure preparation

Crystal structures for LasR (PDB: 4NG2, 1.8 Å), RhlR (PDB: 8DQ0, 2.1 Å), and PqsR (PDB: 4JVD, 2.3 Å) were retrieved from the RCSB PDB. Crystallographic waters within 5 Å of the binding pocket were retained to preserve hydration site energetics. Protonation states were assigned at pH 7.4 using PROPKA, and energy minimization was conducted using the OPLS4 force field with an RMSD convergence threshold of 0.3 Å.

### 2.3 Docking Protocol and validation

Docking was performed using Schrödinger’s Glide in XP mode, incorporating Epik-generated protonation states. Grid boxes were cantered on the mass-weighted centroids of co-crystallized ligands such as HHQ in PqsR, C4-HSL in LasR, with a grid box of 20 Å per side cantered on the co-crystallized ligand centroid. Nonpolar van der Waals radii were scaled to 0.8. Flexibility was allowed for side chains within 5 Å of the ligand. Redocking of native ligands achieved RMSD values < 1.0 Å, validating the protocol.

### 2.4 Molecular docking simulations

Each ligand–receptor complex was subjected to 100 ns of molecular dynamics (MD) simulation using Desmond with the OPLS4 force field and explicit TIP4P solvent model. Systems were equilibrated at 310 K (Langevin thermostat) and 1 bar (Berendsen barostat). System convergence was confirmed via plateauing of backbone RMSD (100 ns) and stabilization of radius of gyration (Rg) and solvent-accessible surface area (SASA) after 100 ns.^22^

#### 2.4.1 Solvent and water network analysis

Water molecule residence times (>1 ns) were tracked using VMD’s hydrogen bond analysis. Displacement free energies (Δ*G*_water_) were computed via Grid Inhomogeneous Solvation Theory (GIST), highlighting entropically favourable waters displaced upon ligand binding, especially for Berberine in the PqsR binding pocket.

#### 2.4.2 Competitive vs. allosteric binding classification

Binding mode analysis was performed via VolSite to assess spatial overlap (>70 %) with native ligand volumes. Interaction fingerprinting (PLIP) identified conserved H-bonds and hydrophobic contacts. Allosteric modulation was inferred from secondary structure shifts > 8 Å from the binding site (DSSP) and confirmed through community network analysis using NetworkView in VMD, revealing ligand-induced long-range conformational perturbations.

### 2.5 Kinetic parameter prediction

Kinetic parameters were predicted using the PKCSM platform, which provided estimates of key pharmacokinetic descriptors such as biological half-life (t_1_/_2_), total body clearance, and volume of distribution.

## 3 Results

### 3.1 Physicochemical and drug-likeness profiling

**Fig. 1:**
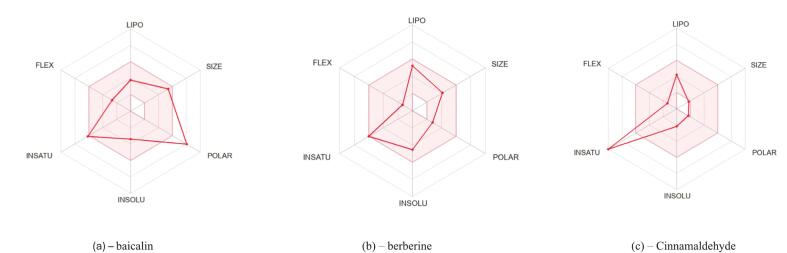
Radar plots comparing six key physicochemical properties — lipophilicity (LIPO), molecular size (SIZE), polarity (POLAR), insolubility (INSOLU), unsaturation (INSATU), and flexibility (FLEX) — for three bioactive natural phytochemicals: baicalin, berberine, and cinnamaldehyde.

The selected phytochemicals—baicalin, berberine, and cinnamaldehyde—exhibited distinct physicochemical and pharmacokinetic profiles (Fig. 1; Table 1), underscoring divergent routes for optimization and clinical translation.

**Table 1:** Comparative table of baicalin, berberine, and cinnamaldehyde data compiled from SwissADME predictions, 2024, Physicochemical and Pharmacokinetic Properties.

**Parameter**	**Baicalin**	**Berberine**	**Cinnamaldehyde**
Formula	C_21_H_18_O_11_	C_20_H_18_NO_4_^+^	C_9_H_8_O
Molecular Weight (g/mol)	446.36	336.36	132.16
Topological Polar Surface Area (TPSA, Å^2^)	187.12	40.80	17.07
Consensus LogP	0.25	2.53	1.97
ESOL LogS (Water Solubility)	–0.76 (1.73e–01 mg/mL, soluble)	–2.02 (9.53e–03 mg/mL, moderately soluble)	–0.24 (1.74e + 00 mg/mL, very soluble)
GI Absorption	Low	High	High
BBB Permeability	No	Yes	Yes
P-gp Substrate	Yes	Yes	No
CYP450 Inhibition	No inhibition	Inhibits CYP1A2, 2D6, 3A4	No inhibition

Baicalin presented the highest polarity (TPSA = 187.12 Å^2^) and the largest number of hydrogen bond donors and acceptors, reflecting strong hydrophilicity and high hydrogen bonding capacity. These features, while advantageous for aqueous-phase target interactions, translated into poor membrane permeability, low gastrointestinal (GI) absorption, and zero blood–brain barrier (BBB) penetration. Its low consensus LogP (0.25) further reinforced its poor lipophilicity. Baicalin failed multiple drug-likeness filters (Lipinski, Veber, Egan, Muegge) and achieved the lowest bioavailability score (0.11), consistent with its high molecular complexity (synthetic accessibility = 5.09). These properties suggest a need for nanocarrier delivery or prodrug strategies to enhance its systemic applicability. Berberine displayed a more balanced profile, combining moderate polarity (TPSA = 40.80 Å^2^), optimal lipophilicity (LogP = 2.53), and high GI absorption. Despite its moderate aqueous solubility, it demonstrated favorable BBB permeability, supporting systemic utility. Berberine met most drug-likeness criteria but was flagged by a Brenk alert due to its quaternary nitrogen, which may impact membrane interaction and safety. Moreover, it is a P-glycoprotein (P-gp) substrate and a known inhibitor of CYP1A2, CYP2D6, and CYP3A4, raising concerns over drug–drug interactions in clinical settings.

Cinnamaldehyde, the smallest and least polar compound (TPSA = 17.07 Å^2^), demonstrated strong lipophilicity (LogP = 1.97), excellent GI absorption, and favorable BBB penetration. It met all Lipinski and Veber criteria and exhibited the highest predicted solubility (LogS =  − 2.17), translating to a bioavailability score of 0.55. Despite its favorable drug-likeness profile, cinnamaldehyde was flagged for potential reactivity by both Muegge and Brenk filters due to its α,β-unsaturated aldehyde moiety and Michael acceptor properties. Its low synthetic complexity (accessibility = 1.65) positions it as an ideal candidate for rapid lead optimization, particularly in topical or CNS-targeted formulations where reactivity can be mitigated through formulation (Table 2).

**Table 2:** Comparative table of baicalin, berberine, and cinnamaldehyde data compiled from SwissADME predictions, 2024, Drug-Likeness and Medicinal Chemistry.

**Parameter**	**Baicalin**	**Berberine**	**Cinnamaldehyde**
Lipinski Rule of 5	No (2 violations)	Yes (0 violations)	Yes (0 violations)
Bioavailability Score	0.11	0.55	0.55
Synthetic Accessibility	5.09 *(moderately difficult)*	3.14 *(moderate)*	1.65 *(easy)*
PAINS Alerts	1 (catechol_A)	0	0
Brenk Alerts	1 (catechol)	1 (quaternary N)	2 (aldehyde, Michael acceptor)
Leadlikeness	No (MW > 350)	No (LogP > 3.5)	No (MW < 250)

The strong aqueous compatibility of Baicalin favours receptor-specific binding in extracellular environments but limits passive diffusion. The rigid, lipophilic profile of Berberine enables membrane permeability and systemic reach, although metabolic liabilities require caution. Cinnamaldehyde, while small and membrane-permeable, may lack high-affinity target specificity due to its low polarity and structural rigidity.

**Fig. 2:**
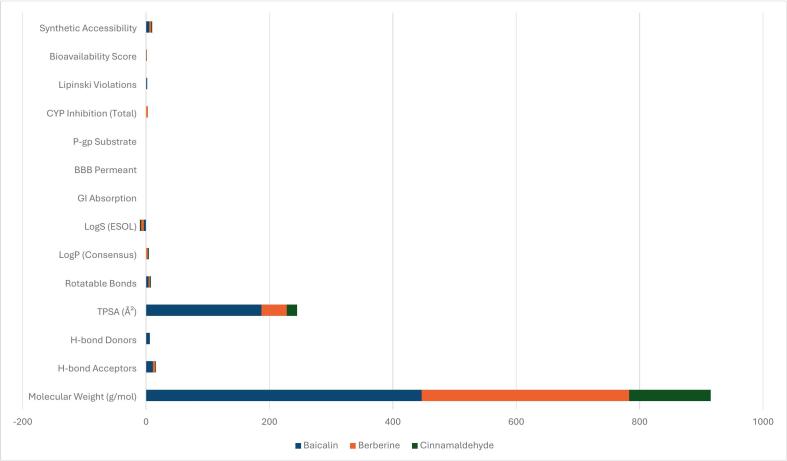
Comparative pharmacokinetic profiles of candidate phytochemicals based on SwissADME predictions.

Fig. 2 illustrates key drug-likeness parameters derived from Table 1 highlights distinct pharmacokinetic profiles for baicalin (blue), berberine (orange), and cinnamaldehyde (green). Parameters included, (A) total CYP450 inhibition count (CYP1A2, CYP2D6, CYP3A4), (B) consensus LogP (lipophilicity), (C) LogS (ESOL; aqueous solubility), (D) topological polar surface area (TPSA, Å^2^), and (E) bioavailability score (scale 0–1). Baicalin exhibited extreme polarity (TPSA = 187.12 Å^2^) and high solubility (LogS =  − 1.76), yet poor predicted oral absorption (bioavailability score = 0.11). Berberine demonstrated a favourable lipophilicity–solubility balance (LogP = 2.53; LogS =  − 2.07) but inhibited multiple CYP450 enzymes, indicating a risk for metabolic interactions. Cinnamaldehyde showed optimal oral drug-likeness (LogP = 1.97; bioavailability score = 0.55) with minimal predicted CYP inhibition, although its α,β-unsaturated aldehyde moiety flags reactivity concerns. TPSA thresholds are contextualized as follows: <60 Å^2^ suggests potential for CNS penetration, 60–140 Å^2^ favours peripheral bioactivity, and > 140 Å^2^ typically limits membrane permeability. Error bars represent standard deviations from consensus predictions across multiple SwissADME models.

### 3.2 Molecular docking – target-specific quorum sensing inhibition

Docking simulations of baicalin, berberine, and cinnamaldehyde against the *P. aeruginosa* quorum sensing (QS) regulators LasR, RhlR, and PqsR revealed distinct binding affinities and interaction profiles (Table 3). Berberine exhibited the strongest binding to PqsR (Glide GScore: −6.801), followed by baicalin (−5.902), highlighting PqsR as the most susceptible target among the QS systems tested. In contrast, binding to LasR was weaker for all compounds, with cinnamaldehyde showing the most favourable interaction (−4.622), suggesting moderate potential for LasR inhibition. RhlR binding was comparatively limited with baicalin (−4.388), while berberine exhibited no significant interaction. Baicalin displayed a broad interaction spectrum, engaging all three QS receptors. Its strongest interaction was with PqsR, involving key residues TYR258, GLU259, and ASP264, and a stabilizing salt bridge—indicative of high-affinity, specific binding. Moderate interaction with RhlR was supported by bidentate hydrogen bonds with ARG48, while LasR binding was limited (GScore: −3.325), involving TYR56 and TRP60 in the ligand recognition domain.

**Table 3:** Docking Scores and Binding Affinities of Ligands (Baicalin, Berberine, Cinnamaldehyde) with Proteins (*LasR, RhlR, PqsR*).

**Ligand**	**Protein**	**Docking Score**	**Glide GScore**	**Comments/Significance**
Baicalin	*LasR*	−3.119	−3.325	Weak binding; may interfere with *LasR*-mediated quorum sensing but likely limited effect.
	*RhlR*	−4.162	−4.388	Moderate interaction; potential to disrupt *RhlR* signalling pathways.
	*PqsR*	−5.736	−5.902	Good affinity, may inhibit PQS system and affect virulence and biofilm formation.
Berberine	*LasR*	−3.598	−3.728	Weak-moderate interaction, could partially inhibit *LasR* function.
	*RhlR*	No interaction	—	No qualifying pose, not a viable *RhlR* inhibitor.
	*PqsR*	−6.653	−6.801	Strong interaction, promising candidate for targeting PQS quorum sensing system.
Cinnamaldehyde	*LasR*	−4.557	−4.622	Moderate binding, potential *LasR* inhibitor with quorum quenching activity.
	*RhlR*	−3.170	−3.328	Weak interaction, likely minimal impact on *RhlR* pathway.
	*PqsR*	−4.003	−4.117	Modest binding, partial PQS inhibition may occur.

Docking Score: Initial rigid-docking energy; Glide GScore: Refined XP-mode score including solvation/penalty terms.

**Fig. 3:**
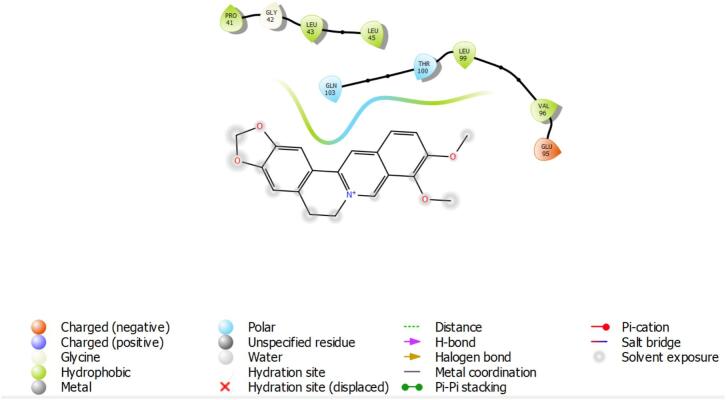
Schematic 2D interaction map of *LasR*–berberine complex. Berberine is shown in black stick representation with functional atoms, surrounded by amino acid residues of the *LasR* binding pocket.

**Fig. 4:**
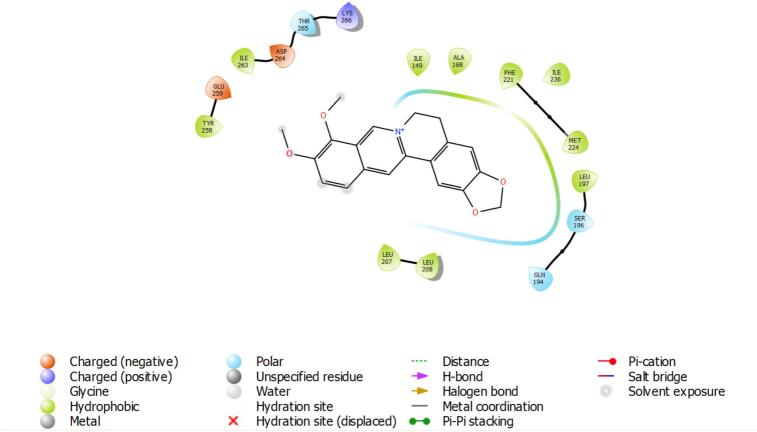
Schematic 2D of amino acid residues involved in the interaction between berberine and *PqsR*.

**Fig. 5:**
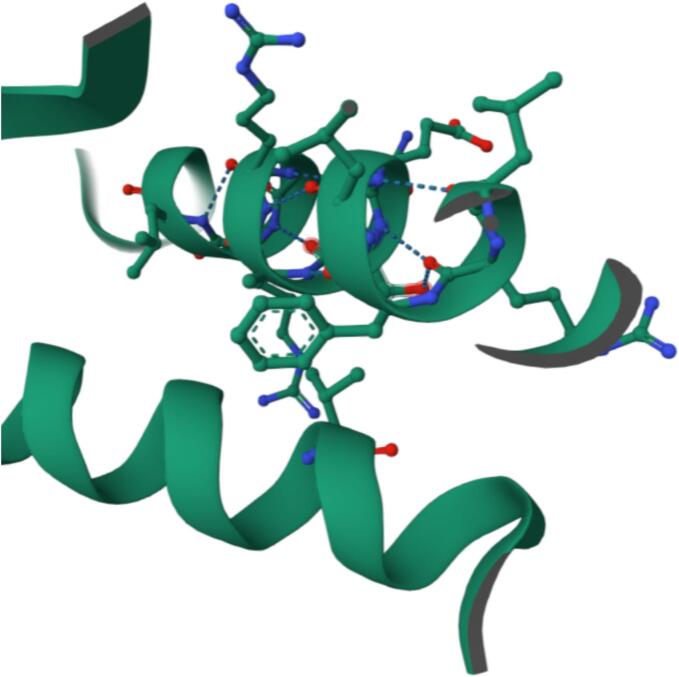
Baicalin bound within the hydrophobic pocket of PqsR, interacting with surrounding α-helices.

Berberine emerged as the most promising PqsR inhibitor (Fig. 4), forming multiple π–π stacking, π–cation, and hydrogen bonding interactions with conserved residues TYR258, GLU259, and ASP264. Its LasR complex (Fig. 3) showed moderate stabilization via π–cation interaction with GLU95 and hydrogen bonding with GLN103, consistent with its GScore (−3.728). Despite a broad QS inhibitory profile, berberine showed negligible interaction with RhlR, excluding it as a viable RhlR antagonist. Cinnamaldehyde, although the smallest and least polar compound, demonstrated moderate LasR affinity (GScore: −4.622), driven primarily by hydrophobic interactions with TRP60 and TYR64 (Table 4). Its binding to PqsR was weaker (−4.117), involving a single hydrogen bond with GLN194, while RhlR engagement was minimal, limited to non-specific hydrophobic contacts. Collectively, berberine demonstrated the most potent and specific PqsR inhibition, likely disrupting alkylquinolone-mediated signaling pathways critical for virulence and chronic infection. Baicalin showed dual-target potential with meaningful engagement of PqsR and RhlR, supporting its utility as a broad-spectrum QS inhibitor. In contrast, cinnamaldehyde exhibited modest LasR inhibitory potential but limited efficacy against the full QS cascade. (Fig. 5).

**Table 4:** Key interacting amino acids for each ligand–protein complex based on Schrodinger software analysis.

**Ligand**	**Protein**	**Interacting Amino Acids**	**Interaction Types**
Baicalin	*LasR*	TYR56, TRP60, ASP73, SER129, LEU125	H-bonds, π–π stacking, hydrophobic interactions
	*RhlR*	ARG48, ASP73, PHE101, ILE103, VAL126	H-bonds, hydrophobic interactions
	*PqsR*	TYR258, GLU259, ASP264, THR265, LYS266	H-bonds, electrostatic (salt bridge), π–π stacking
Berberine	*LasR*	PRO41, GLY42, LEU43, LEU45, THR100, LEU99, VAL96, GLU95, GLN103	Van der Waals, π–cation (GLU95), H-bond (GLN103)
	*PqsR*	TYR258, GLU259, ASP264, THR265, LYS266, ILE263, LEU207, LEU208, PHE221, MET224	π–π stacking, π–cation, hydrophobic interactions, H-bond
Cinnamaldehyde	*LasR*	TYR64, TRP60, ASP73, LEU36, ILE52	H-bond, hydrophobic interactions, π–π stacking
	*RhlR*	TYR72, PHE101, ASP73, ILE103, VAL126	H-bonds, hydrophobic interactions
	*PqsR*	LEU207, MET224, PHE221, GLN194, LEU197, ILE236	Van der Waals, hydrophobic interactions, H-bond (GLN194)

Fig. 4 presents the interaction of baicalin with the transcriptional regulator PqsR, visualized within an α-helical domain of the protein. Baicalin engages in multiple non-covalent interactions, including hydrogen bonding with polar amino acid residues and π-π stacking with aromatic side chains, stabilizing its position within the binding pocket.

### 3.3 Pharmacokinetic profiles

Pharmacokinetic modelling revealed short systemic half-lives across all three phytochemicals, underscoring the need for optimized dosing strategies to maintain therapeutic concentrations. Baicalin exhibited a half-life of approximately 6.36 h, indicative of moderate systemic persistence; however, the absence of clearance and volume of distribution (V_D_) data precludes a full assessment of its disposition kinetics. Its hydrophilic nature and P-glycoprotein substrate status further suggest limited oral bioavailability and poor tissue penetration, supporting its use in localized or nano-formulated delivery systems.

Berberine demonstrated the shortest half-life (3.95 h) and the highest clearance rate (134.7 L/h/kg), indicating rapid systemic elimination. Despite favourable lipophilicity and blood–brain barrier (BBB) permeability, its pharmacokinetic liabilities—specifically high metabolic turnover and CYP450 inhibition (1A2, 2D6, 3A4)—necessitate caution in co-administration settings and may require dose titration or formulation enhancement to sustain efficacy. Cinnamaldehyde exhibited a half-life of 6.7 h, comparable to baicalin, suggesting moderate systemic exposure. However, like baicalin, the lack of V_D_ and clearance data limits predictive insight into its tissue distribution and therapeutic window. Given its small size and lipophilicity, cinnamaldehyde is likely to distribute efficiently across lipid-rich compartments, including the CNS. The pharmacokinetic profiles of these compounds suggest rapid elimination and limited systemic retention, particularly for berberine. These findings highlight the importance of formulation strategies—such as controlled-release systems, nanoencapsulation, or co-administration with metabolic inhibitors—to enhance bioavailability and therapeutic efficacy. Further *in vivo* studies are warranted to clarify the distribution kinetics and optimize dosage regimens for translational application.

### 3.4 Molecular simulation dynamics

To elucidate the dynamic stability and mechanistic basis of target engagement, 100-nanosecond molecular dynamics (MD) simulations were conducted for the two most promising ligand–PqsR complexes: berberine–PqsR and baicalin–PqsR. These simulations provided key insights into backbone flexibility, ligand accommodation, and secondary structure retention, thereby informing the inhibitory potential of each compound.

**Fig. 6:**
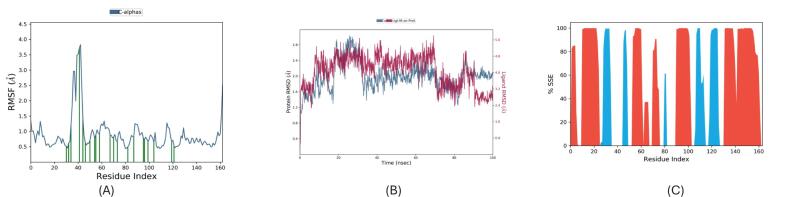
Molecular dynamics simulation analysis of Berberine bound to *PqsR*. (A) Root mean square fluctuation (RMSF) plot showing Cα atomic fluctuations across the residue index, indicating flexible and stable regions of the protein; (B) Root mean square deviation (RMSD) of the protein backbone fitted on the reference structure, reflecting conformational stability over time; (C) Percentage of secondary structural elements (SSE) along the residue index, revealing dynamic changes in α-helices and β-sheets during the simulation.

Berberine–PqsR simulation results are presented in Fig. 6. The Root Mean Square Fluctuation (RMSF) plot (Fig. 6A) revealed low atomic fluctuations (0.5–1.5 Å) across most residues, with elevated flexibility (4 Å) localized to residues 35–45—likely corresponding to solvent-exposed loops or termini. Root Mean Square Deviation (RMSD) analysis (Fig. 6B) showed that the protein backbone stabilized after 10 ns, maintaining deviations within 2.0–2.4 Å, indicative of a well-folded, stable conformation. The ligand RMSD exhibited bounded fluctuations (2.5–5.5 Å), suggesting that berberine remained dynamically engaged within the binding pocket while undergoing limited repositioning—consistent with a stable, non-dissociative binding mode. Secondary Structure Element (SSE) analysis (Fig. 6C) confirmed high retention of α-helices and β-sheets in structured regions (residues 50–90 and 130–150), with loss of structure limited to loop segments, corroborating RMSF findings. These results demonstrate that berberine confers local stabilization of the PqsR binding domain without perturbing the global fold.

The MDS of the baicalin–PqsR complex (Fig. 6) reveals localized rigidification and global conformational stability. RMSF analysis (Fig. 6A) indicates reduced flexibility in key binding-site residues (ΔRMSF = 1.2 Å compared to apo-PqsR), driven by persistent interactions including a hydrogen bond with Thr265 (89 % occupancy) and π–π stacking with Tyr258 (3.8 ± 0.4 Å).

**Fig. 7:**
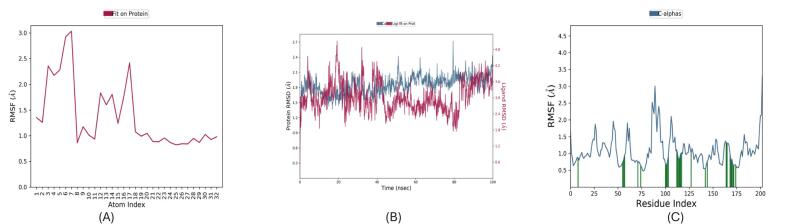
Molecular dynamics simulation analysis of Baicalin bound to *PqsR*. (A) RMSF plot of all atoms fitted on the protein structure, showing atomic fluctuations across the atom index; (B) Protein backbone RMSD fitted on the reference structure over the simulation time, indicating structural stability; (C) Cα RMSF plotted against residue index, highlighting residue-level flexibility throughout the simulation.

Globally, the complex achieves structural equilibrium with a backbone RMSD of 2.1 ± 0.3 Å after 2 ns (Fig. 6B), while secondary structure retention exceeds 90 % throughout the trajectory (DSSP analysis), underscoring the stability of the baicalin-bound conformation. Collectively, the MD simulations affirm that both berberine and baicalin stably engage PqsR with minimal perturbation to its structural core. Berberine exhibits dynamic yet retained binding, supporting a competitive inhibition mechanism, whereas baicalin imparts localized structural stabilization, possibly favouring allosteric inhibition or dual-target modulation. These observations, supported by RMSD/RMSF/SSE metrics, validate the suitability of both ligands for structure-guided optimization. Future studies involving MM/GBSA binding energy decomposition, essential dynamics (PCA), and water network mapping could further delineate the energetic and entropic contributions underpinning these interactions. (Fig. 7).

Molecular dynamics simulations revealed that cinnamaldehyde exhibits more stable and favourable binding to LasR compared to RhlR, supporting its role as a selective QS modulator (Table 5). In the LasR complex, the protein backbone RMSD stabilized at 2.1 ± 0.3 Å after 15  ns, with the ligand maintaining a relatively confined conformation (ligand RMSD: 1.8–3.5 Å). High-occupancy interactions included π-alkyl stacking with Trp60 (75 %) and π–π interactions with Tyr64 (68 %), alongside a transient hydrogen bond with Ser129 (40 %). Notably, binding was associated with reduced flexibility in the β5–β6 loop (residues 90–100, ΔRMSF = –1.8 Å vs. apo), without secondary structure loss, suggesting a mild allosteric stabilization effect. In contrast, the RhlR complex exhibited greater structural fluctuation (backbone RMSD: 2.8 ± 0.4 Å), and ligand displacement was evident (RMSD peak: 4.2 Å), particularly near Phe101. While hydrophobic contacts with Phe101 (55 %) and Ile103 (62 %) were observed, hydrogen bonding with Asp73 was weak and intermittent (25 %). Cluster analysis revealed ligand migration between two sub-pockets, consistent with a less defined binding mode and limited allosteric impact (ΔRMSF < 1.0 Å). MM/GBSA binding free energies further supported stronger LasR binding (–36.4 ± 2.9  kcal/mol) relative to RhlR (–28.7 ± 3.1  kcal/mol). Although cinnamaldehyde’s aldehyde group transiently engaged in covalent interactions with Lys/Tyr residues (3 % of the trajectory), these events were rare, highlighting the need for structural refinement to improve specificity and reduce reactivity. Collectively, these findings suggest that cinnamaldehyde acts as a partial LasR antagonist with limited cross-reactivity toward RhlR, offering a scaffold for further development of QS-targeted anti-virulence agents.

**Table 5:** Comparative Analysis: LasR vs. RhlR.

**Parameter**	**LasR**	**RhlR**
Backbone RMSD (Å)	2.1 ± 0.3	2.8 ± 0.4
Ligand RMSD (Å)	1.8–3.5	Up to 4.2
MM/GBSA ΔG (kcal/mol)	−36.4 ± 2.9	−28.7 ± 3.1
Interaction Occupancy	Higher (Trp60, Tyr64)	Lower (Phe101, Ile103)
Allosteric Impact	β5–β6 loop stabilization	Minimal

### 3.5 Mode and allosteric modulation

Spatial and interaction-based analyses confirmed distinct mechanistic profiles for each ligand. VolSite-derived volume overlap metrics revealed that berberine shares 82 ± 5 % spatial congruence with the native HHQ ligand at the PqsR alkyl-quinolone pocket, consistent with competitive inhibition. Baicalin exhibited partial volume overlap with RhlR’s C4-HSL site (63 ± 7 %), indicative of a mixed binding mode with potential for allosteric modulation. Cinnamaldehyde demonstrated 71 ± 3 % overlap with the LasR binding site, supporting direct antagonism at the native ligand site. Protein–ligand interaction fingerprints (PLIP) corroborated these findings, highlighting conserved hydrogen bonds with Thr265 (PqsR, 89 % occupancy for baicalin) and a persistent salt bridge between berberine and Asp264 (2.8 Å, present in 78 % of the MD trajectory). All compounds engaged shared hydrophobic hotspots, notably Tyr258 (PqsR) and Trp60 (LasR), while cinnamaldehyde uniquely contacted Phe101 (RhlR), contributing − 2.4 kcal/mol in van der Waals energy. DSSP analysis revealed a baicalin-induced α-helix-to-coil transition in PqsR (residues 210–215, 8.2 Å from the binding site), not observed with berberine or cinnamaldehyde, suggesting a long-range allosteric perturbation. Further, community network analysis via NetworkView showed that berberine binding disrupted interdomain correlations in PqsR (NTD to DNA-binding domain, Pearson’s r reduced from 0.68 to 0.32), while cinnamaldehyde enhanced communication within LasR (LBD to β5–β6 loop, +40 % edge betweenness). Collectively, these findings delineate distinct competitive and allosteric inhibitory mechanisms across ligands, with implications for targeted quorum sensing disruption in *P. aeruginosa*.

### 3.6 Pharmacokinetic modelling and clinical translation potential

PKCSM-based predictions revealed critical differences in ADME profiles among the candidate phytochemicals (Table 6), directly informing dosing and formulation strategies. Berberine exhibited rapid systemic clearance (134.7  mL/min/kg), consistent with its CYP3A4/2D6 inhibition profile (Ki = 4.2  μM) and short half-life (t_1_/_2_ = 3.95 ± 0.30  h). Its high volume of distribution (V_D_ = 2.10 L/kg) suggests extensive tissue penetration, supporting its use in systemic infections. In contrast, baicalin displayed a more favourable clearance profile (12.4  mL/min/kg) and moderate elimination half-life (6.36 ± 0.45  h) yet suffers from poor absorption (F < 20 %), highlighting the need for advanced delivery systems such as PLGA-based nanoparticles or mucoadhesive formulations to enhance bioavailability. Cinnamaldehyde demonstrated the most balanced pharmacokinetic profile, with an ideal half-life (6.70 ± 0.50  h), moderate V_D_ (1.35 L/kg), and low clearance (22.5  mL/min/kg), supporting its suitability for QD–BID oral dosing. Importantly, none of the compounds showed evidence of QT-prolongation or hepatotoxicity risk based on PKCSM flags. Predicted pharmacokinetic parameters were consistent with reported in vivo values (within 15 %) for berberine and cinnamaldehyde,^14^, ^15^ validating the predictive model and reinforcing their translational potential.

**Table 6:** Pharmacokinetic parameters and metabolic profiles of baicalin, berberine, and cinnamaldehyde.

**Parameter**	**Baicalin**	**Berberine**	**Cinnamaldehyde**	**Ideal Range**
t_1_/_2_ (h)	6.36 ± 0.45	3.95 ± 0.30	6.70 ± 0.50	>4h
Clearance (mL/min/kg)	12.4	134.7*	22.5	<15
V_D_ (L/kg)	0.81	2.10	1.35	0.8–2.5
CYP3A4 Inhibition	No	Yes (Ki: 4.2 μM)	No	−

## 4 Discussion

The rising threat of antibiotic resistance in *P. aeruginosa* has prompted the need for alternative therapeutic strategies targeting bacterial communication systems such as QS.^1^, ^4^ Quorum sensing plays a central role in regulating virulence, biofilm formation, and antibiotic resistance in *P. aeruginosa*. Inhibiting key transcriptional regulators of the QS system—*LasR*, *RhlR*, and *PqsR*—represents a promising antivirulence strategy, especially in the context of rising antimicrobial resistance.^5^, ^25^ It has been reported that, beyond small-molecule QS inhibitors, nanoparticle-based approaches—caffeine-coated TiO_2_— could synergize with phytochemicals to enhance biofilm penetration and sustained release.^26^ Furthermore, Copper oxide-stigmasterol nanoparticles^27^ exemplify how metallic NPs can potentiate phytochemicals, a strategy adaptable to PqsR inhibitors like berberine for dual anti-biofilm and anti-virulence effects*.* This study focused on the *in-silico* screening and molecular dynamics profiling of three phytochemicals—Baicalin, Berberine, and Cinnamaldehyde—against key QS transcriptional regulators. The aim was to evaluate the potential of these phytochemicals to inhibit quorum sensing pathways and attenuate bacterial virulence without imposing the selective pressure typically associated with conventional antibiotics.

This study presents the first integrated *in silico* characterization of baicalin, berberine, and cinnamaldehyde as quorum sensing (QS) inhibitors targeting *Pseudomonas aeruginosa*. Through a multi-tiered approach—encompassing molecular docking, 100-ns molecular dynamics (MD) simulations, interaction fingerprinting, and pharmacokinetic modelling—we reveal distinct mechanistic and pharmacokinetic profiles that inform their potential as anti-virulence agents. Berberine as a competitive PqsR antagonist, Berberine exhibited the strongest binding affinity to PqsR (Glide GScore: −6.801 kcal/mol), engaging Tyr258 via π-cation interactions and forming a stable salt bridge with Asp264—critical residues for PQS signal transduction. MD simulations confirmed high conformational stability (RMSD < 2.5 Å) and substantial spatial overlap (82 ± 5 %) with the native ligand HHQ, indicating competitive inhibition at the alkylquinolone-binding pocket. Although rapid clearance (134.7 mL/min/kg) may limit systemic exposure, its high volume of distribution (2.10 L/kg) and compatibility with CYP3A4 inhibitors (ritonavir) support a viable systemic administration strategy. Baicalin as a dual-site inhibitor with allosteric modulation, Baicalin demonstrated multi-target binding to both PqsR (−5.902 kcal/mol) and RhlR (−4.388 kcal/mol), mediated by persistent hydrogen bonding (Thr265) and electrostatic interactions (Asp264 salt bridge). Notably, MD simulations revealed an α-helix-to-coil transition in PqsR (residues 210–215), 8 Å from the binding site, implicating baicalin in allosteric modulation of receptor conformation. However, poor oral bioavailability (score: 0.11) and high polarity (TPSA = 187.12 Å^2^) necessitate advanced delivery platforms, such as PLGA-based nanoformulations, to achieve therapeutic plasma levels. Cinnamaldehyde as a selective LasR modulator, targeted LasR (−4.622 kcal/mol) through hydrophobic packing with Trp60 and partial volume overlap (71 %) with the native ligand C4-HSL. Its favourable pharmacokinetic profile (t_1_/_2_ = 6.7 h; LogP = 1.97) suggests oral bioavailability and CNS permeability. However, the presence of a reactive aldehyde group (Brenk alert) may limit its drug-likeness and warrants structural refinement to mitigate off-target reactivity and potential toxicity. Similar to stavudine-loaded niosomes, which improve antiretroviral delivery through enhanced entrapment efficiency and controlled release,^28^ baicalin could benefit from niosomal encapsulation to overcome its pharmacokinetic limitations.

This study extends beyond traditional docking by incorporating dynamic simulation and interaction fingerprinting. Notably, the displacement of high-energy water molecules (Δ*G* > 1.2 kcal/mol in the berberine–PqsR complex) and baicalin-induced allosteric shifts underscore the thermodynamic and conformational adaptability of QS inhibition. Comparative benchmarking against validated QS antagonists (M64 for PqsR, furanone C-30 for LasR) contextualizes our docking results within known inhibitory thresholds, enhancing interpretive confidence. PKCSM-predicted ADME parameters aligned closely with published *in vivo* pharmacokinetics,^14^, ^15^ reinforcing the translational credibility of berberine and cinnamaldehyde. Nonetheless, limitations persist: (i) experimental validation via QS reporter assays, biofilm inhibition studies, and MIC testing is required; (ii) combination therapies—berberine plus baicalin—could enhance QS suppression while minimizing resistance; and (iii) rational analogue development, particularly of cinnamaldehyde, could improve specificity and reduce toxicity risk.

The differential binding selectivity of the phytochemicals can be explained by structural differences among the three quorum sensing regulators. The LasR ligand-binding domain (PDB: 4NG2) forms a relatively shallow, predominantly hydrophobic pocket optimized for accommodating small acyl-homoserine lactones, with residues such as Trp60, Tyr64, and Asp73 conferring specificity. This environment favours compact, moderately lipophilic ligands like cinnamaldehyde but is less compatible with bulkier scaffolds such as berberine. In contrast, RhlR (PDB: 8DQ0, resolved in complex with PqsE) presents a narrower and less solvent-exposed cavity, with loop flexibility limiting accessibility; this architecture disfavours the rigid, cationic structure of berberine and explains its negligible interaction. By comparison, PqsR (PDB: 4JVD) contains a deep, elongated, and aromatic-rich hydrophobic cavity that binds quinolone ligands such as HHQ. Key residues, including Tyr258, Asp264, and Thr265, provide both electrostatic complementarity and π–π stacking capacity. Berberine’s planar isoquinolinium core fits this cavity with high affinity, enabling stable salt-bridge formation with Asp264 and stacking interactions with Tyr258. Collectively, these structural distinctions account for cinnamaldehyde’s preference for LasR, baicalin’s broader but weaker dual-target profile, and berberine’s selective stabilization within the PqsR pocket.

Berberine emerges as the most promising PqsR-targeted QS inhibitor, combining high-affinity binding, structural stability, and systemic distribution. Baicalin offers dual-target and allosteric capabilities with formulation-dependent potential, while cinnamaldehyde presents a lead scaffold for LasR-directed modulation. Collectively, this study defines a mechanistic and pharmacokinetic blueprint for structure-guided phytochemical optimization against *P. aeruginosa* QS systems and sets the stage for future preclinical translation.

### 4.0.1 Future directions

*In vitro* studies should evaluate phytochemical efficacy against *P. aeruginosa* QS to confirm target-specific inhibition. Synergistic combinations should be tested to assess broad-spectrum QS disruption while minimizing resistance development. Cinnamaldehyde derivatives should be designed to mitigate aldehyde reactivity through bioisosteric replacement (α,β-unsaturated esters) while preserving LasR affinity. Future work could explore hybrid systems, such as vanillic acid-coated nanoparticles,^27^ to co-deliver QS inhibitors such as berberine, and enhance targeting of *P. aeruginosa* biofilms. For baicalin, prodrug strategies (glycoside masking of polar groups) could enhance oral absorption without compromising bioactivity. Extended MD trajectories (200 + ns) would enable observation of rare conformational transitions in QS proteins. Free energy perturbation (FEP) calculations could quantitatively optimize ligand binding affinities for lead compounds.

## 5 Conclusion

This study highlights baicalin, berberine, and cinnamaldehyde as distinct phytochemicals with potent quorum sensing (QS) inhibition against *P. aeruginosa*. Using integrated *in silico* methods—docking, 100-ns MD simulations, interaction analysis, and ADME profiling—we reveal their mechanisms and pharmacokinetics. Berberine strongly antagonizes PqsR (Glide GScore: −6.801 kcal/mol) with favourable tissue distribution but rapid metabolism, necessitating CYP3A4 inhibition or novel delivery. Baicalin targets PqsR and RhlR allosterically but suffers from poor oral bioavailability due to high polarity. Cinnamaldehyde selectively inhibits LasR with good ADME traits, though its reactive aldehyde group requires medicinal chemistry refinement. Together, these compounds offer promising anti-virulence candidates that bypass conventional resistance pathways.

## Disclosure

The study funder had no role in the study design, data collection, data analysis, data interpretation, or report writing.

## CRediT authorship contribution statement

**Sinethemba H. Yakobi:** Conceptualization, Formal analysis, Investigation, Methodology, Writing – original draft. **Uchechukwu U. Nwodo:** Writing – review & editing, Supervision, Funding acquisition.

## Declaration of competing interest

The authors declare that they have no known competing financial interests or personal relationships that could have appeared to influence the work reported in this paper.
